# Supplementary material for: A new paradigm of islet adaptations in human pregnancy: insights from immunohistochemistry and proteomics
Source: Nat Commun. 2025 Jul 21;16:6687. doi: 10.1038/s41467-025-61852-5 (PMC12280027; doi:10.1038/s41467-025-61852-5)
Supplement: Supplementary file 1 — Supplementary Information [file 41467_2025_61852_MOESM1_ESM.pdf]

SUPPLEMENTARY

Supplementary Figures and Tables

a

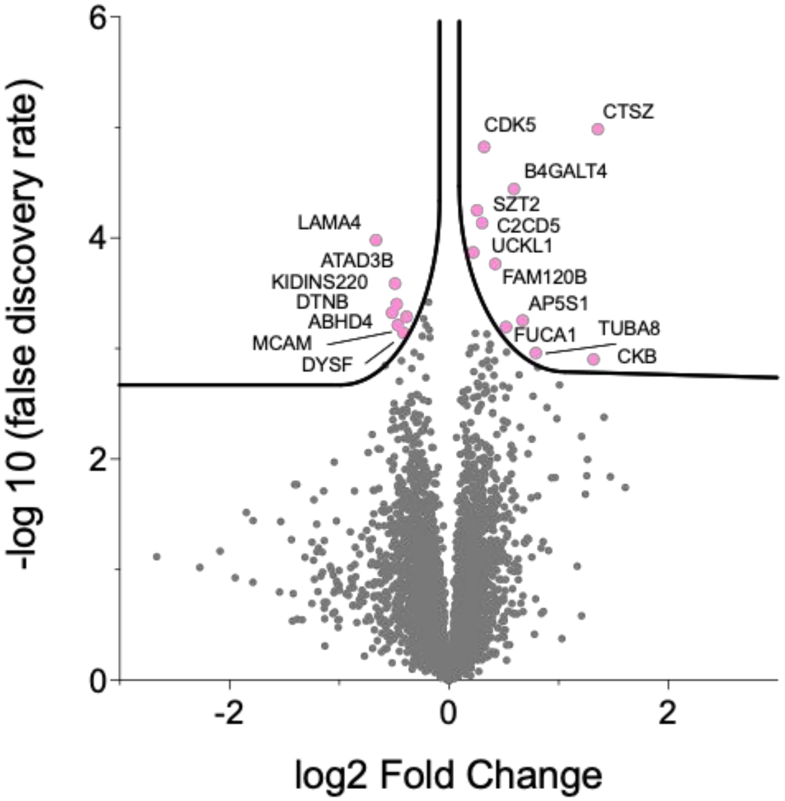

b

Gene name	Protein name	log2 FC	-log 10 FDR
B4GALT4	Beta-1,4-galactosyltransferase 4	0.59	4.44
DTNB	Dystrobrevin beta	-0.52	3.32
DYSF	Dysferlin	-0.47	3.21
FUCA1	Tissue alpha-L-fucosidase	0.52	3.20
CKB	Creatine kinase B-type	1.32	2.90
MCAM	Cell surface glycoprotein MUC18	-0.48	3.40
CDK5	Cyclin-dependent-like kinase 5	0.32	4.82
LAMA4	Laminin subunit alpha-4	-0.66	3.98
SZT2	KICSTOR complex protein SZT2	0.26	4.25
ATAD3B	ATPase family AAA domain-containing protein 3B	-0.49	3.59
C2CD5	C2 domain-containing protein 5	0.30	4.13
ABHD4	Lyso-N-acylphosphatidylethanolamine lipase	-0.41	3.14
FAM120B	Constitutive coactivator of peroxisome proliferator-activated receptor gamma	0.42	3.77
AP5S1	AP-5 complex subunit sigma-1	0.67	3.25
UCKL1	Uridine-cytidine kinase-like 1	0.22	3.87
TUBA8	Tubulin alpha-8 chain	0.79	2.96
CTSZ	Cathepsin Z	1.36	4.98
KIDINS220	Kinase D-interacting substrate of 220 kDa	-0.39	3.29

Supp. Fig. S1: Exploratory analysis of protein expression of islets from pregnant women.
a Volcano plot depicting differentially expressed proteins in islets from pregnant women compared to non-pregnant controls. Significantly expressed proteins are shown in pink and are labelled. Statistical significance was determined using an FDR < 0.15 (corresponding to –log 10 FDR > 0.82) and S0 = 0.01. **b** A table listing differentially abundant proteins identified using an FDR < 0.15, with corresponding log 2-fold change (FC) and –log 10 FDR values.

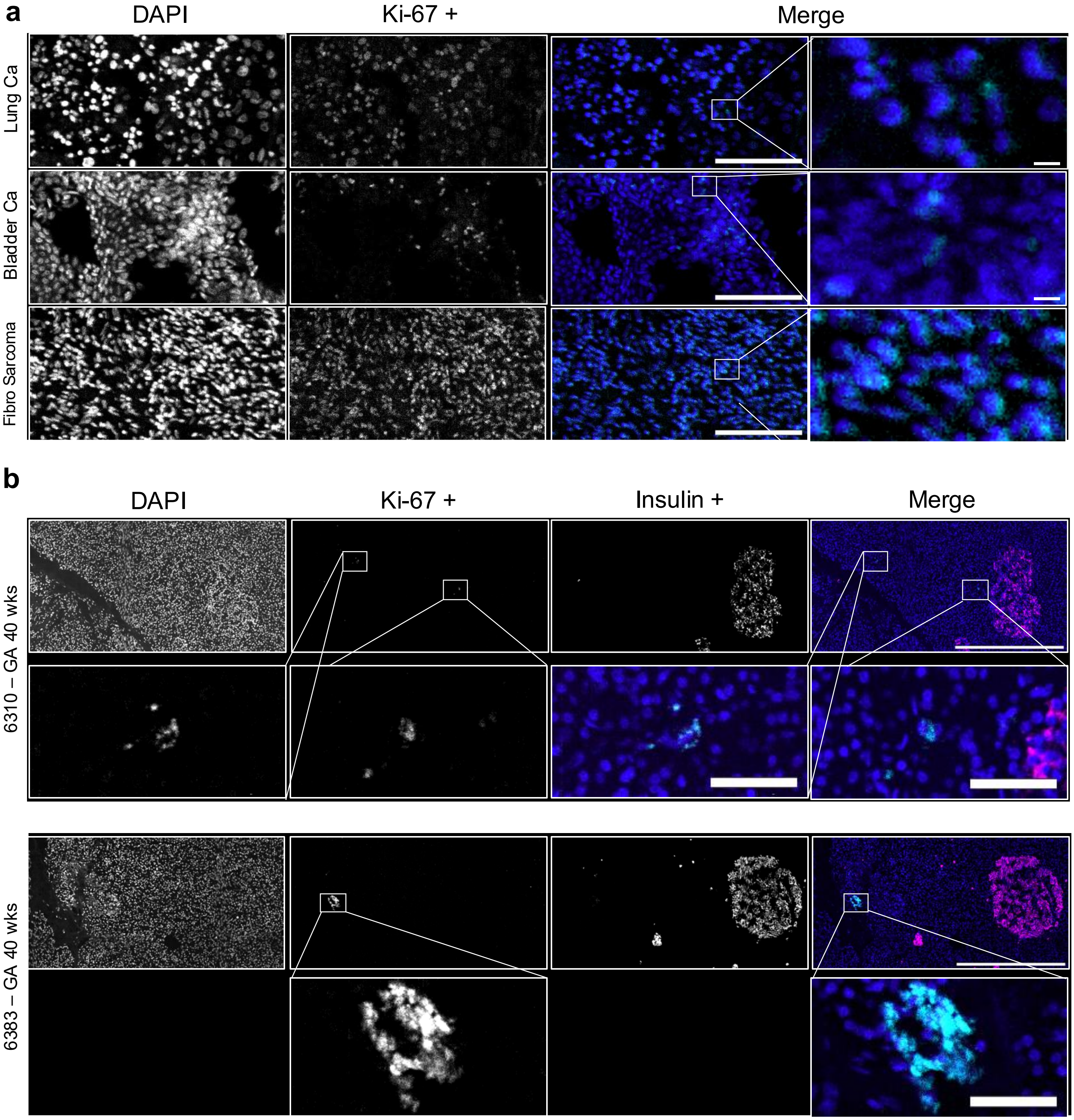

Supp Fig. S2: Positive controls for anti-Ki67 antibody used for immunofluorescence (IHC-IF). **a** IHC-IF labelling of lung carcinoma (squamous cell carcinoma), bladder carcinoma (papillary transitional cell carcinoma), and fibrosynovial sarcoma for Ki-67 with the anti-Ki67 antibody (Table S2). Grayscale images of the nuclei (DAPI) and Ki-67 + channels are shown, and the merged composite image shows the nuclei (DAPI, blue) and Ki-67 positive nuclei (cyan). Scale bar = 100 μm. A magnified inset image from the merged channel is also shown, Scale bar = 5 μm. **b** Anti-Ki-67 labelling of human pancreatic sections from pregnant women at 40 weeks. Ki-67 + exocrine cells are identified in the pancreatic sections in which islets were analysed for the presence of Ki67. Grayscale images of the nuclei (DAPI), Ki-67 +, and insulin + channels are shown, and the merged composite image shows the nuclei (DAPI, blue), Ki-67 + nuclei (cyan), and insulin (magenta). Scale bar = 500 μm and a magnified inset image from the Ki-67+ grayscale channel and merged image is also shown, Scale bar = 50 μm. Representative images from two donors are shown; similar results were observed across the 14 biological replicates, each representing an independent human donor.

*GA – gestational age, wks – weeks, Ca - carcinoma.

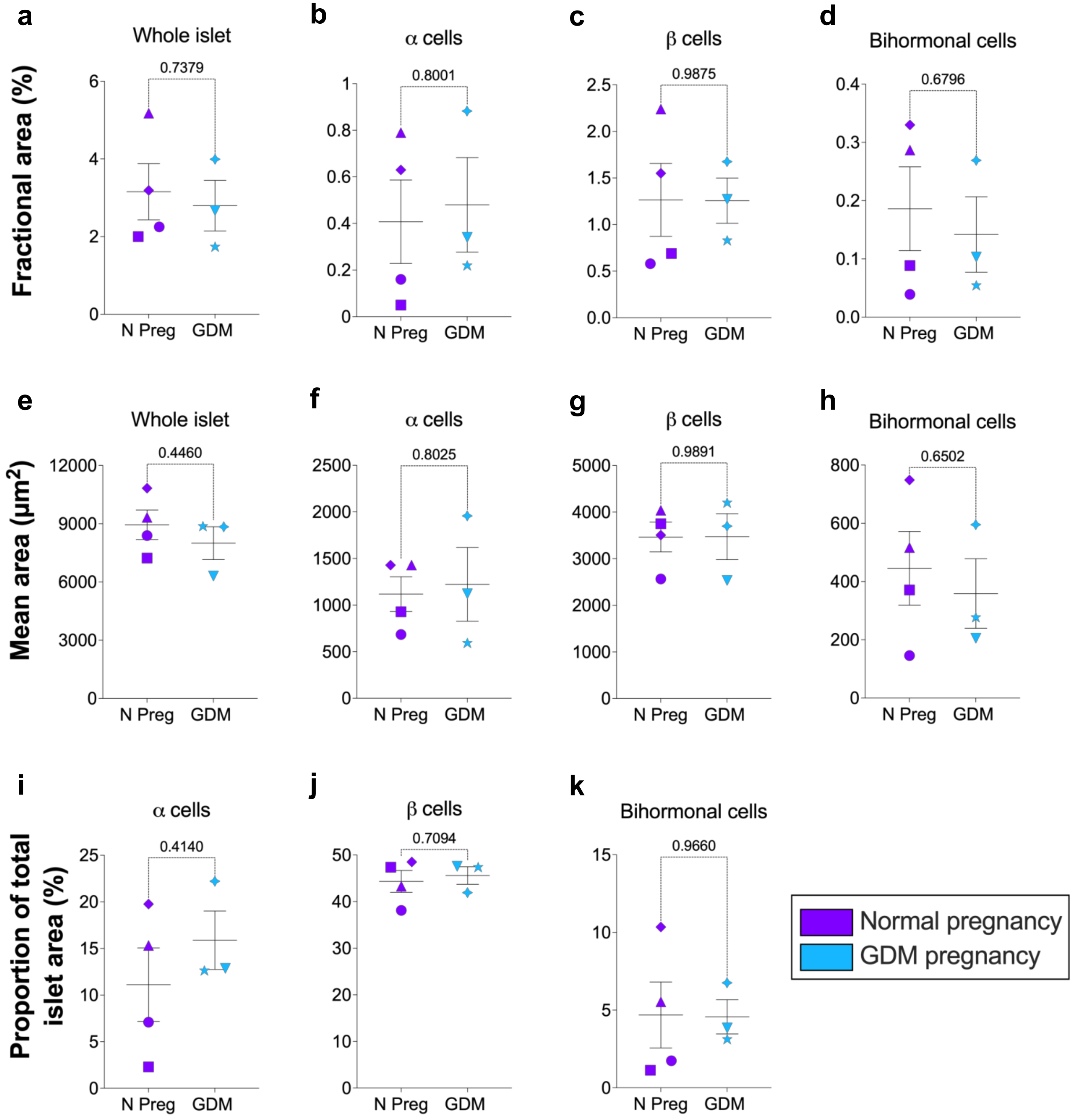

Supp. Fig. S3: Quantitative comparisons of islet metrics between normal and gestational diabetes (GDM) pregnancy.
a-j The data includes comparisons for **a-d** fractional and **e-h** mean areas of whole islets, α cells, β cells, and bihormonal cells. Additionally, comparisons of the **i-k** proportions of α, β, and bihormonal cells relative to whole islets are shown. Symbols in each figure correspond to individual donors as indicated in Table 1. The normal pregnancy group (n = 4 biological replicates) was compared to the GDM pregnancy group (n = 3 biological replicates). Each biological replicate represents an independent human donor. Data are presented as mean ± SEM. Normally distributed data were analysed using a two-sided unpaired Student's t-test; non-parametric data were analysed using a two-sided Mann–Whitney test. Exact p-values for each comparison are shown in the figure. Statistical significance was defined as $P < 0.05$.

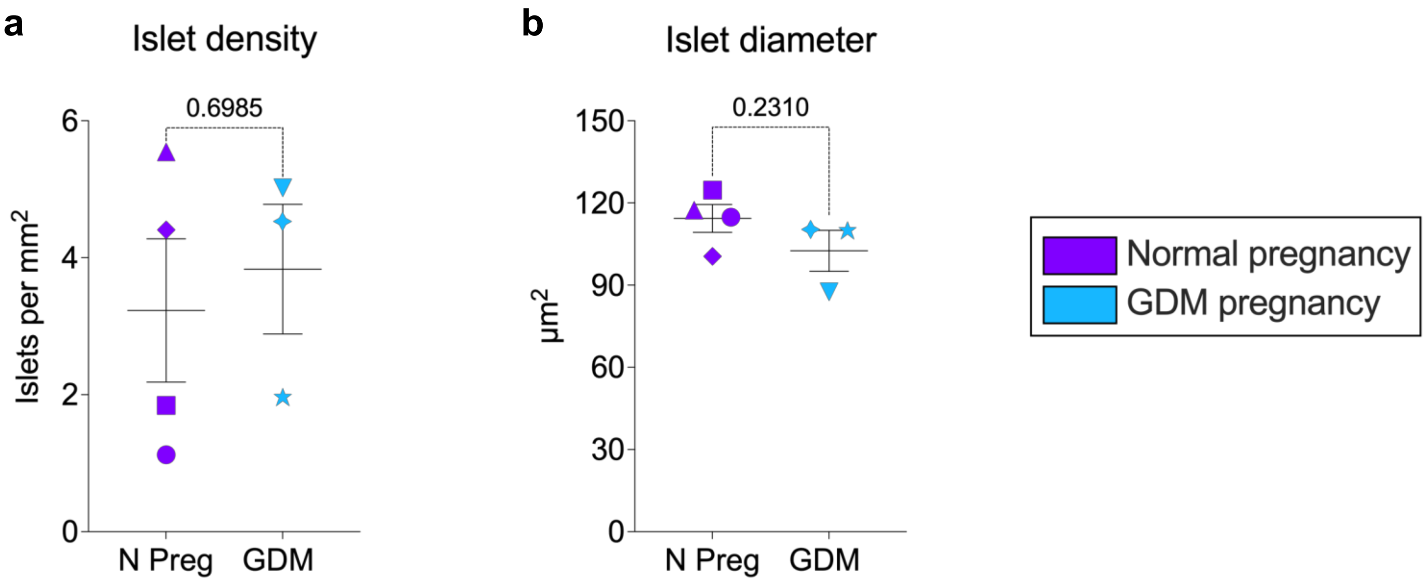

Supp. Fig. S4: Islet density and whole islet diameter compared between normal and gestational diabetes (GDM) pregnancy.

a Islet density and **b** islet diameter. Symbols in each figure correspond to individual donors as indicated in Table 1. The normal pregnancy group (n = 4 biological replicates) was compared to the GDM pregnancy group (n = 3 biological replicates). Each biological replicate represents an independent human donor. Data are presented as mean ± SEM. Normally distributed data were analysed using a two-sided unpaired Student's t-test; non-parametric data were analysed using a two-sided Mann–Whitney test. Exact p-values for each comparison are shown in the figure. Statistical significance was defined as $P < 0.05$.

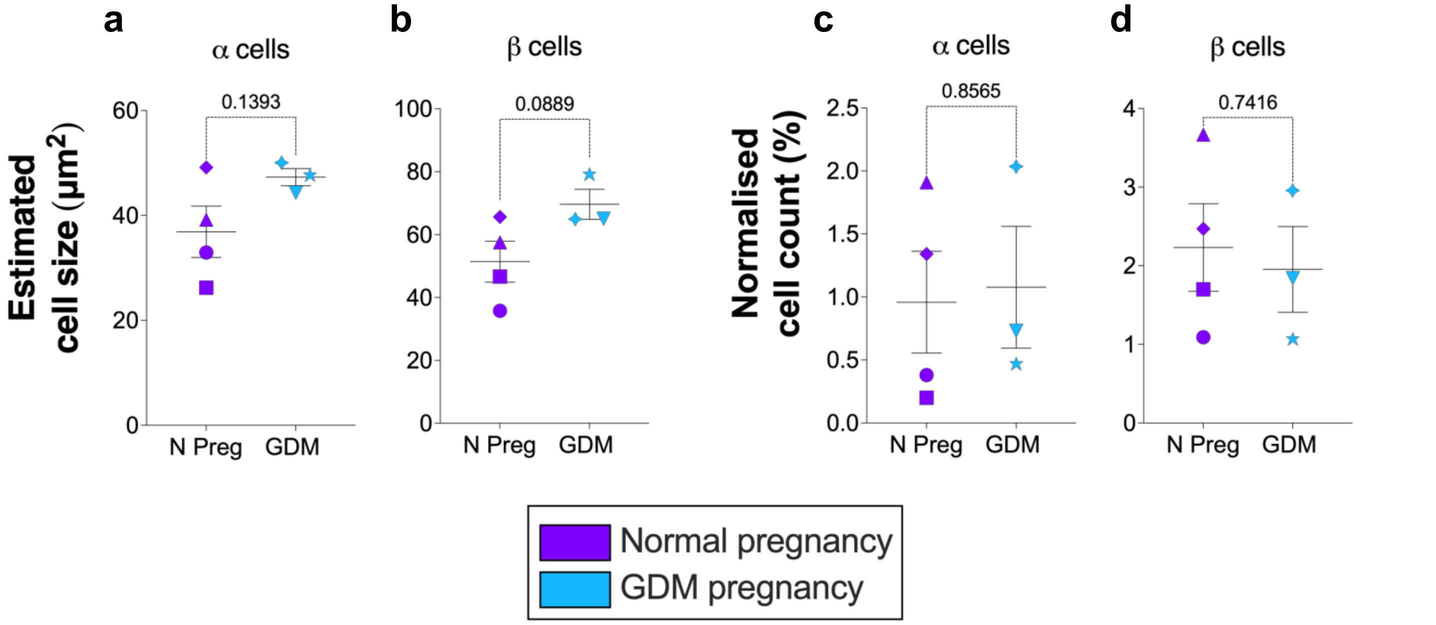

Supp. Fig. S5: Quantitative comparisons of estimated cell size and normalised cell count between Normal and gestational (GDM) pregnancy.

Measurements of estimated **a** α-cell and **b** β-cell sizes, as well as normalised **c** α-cell and **d** β-cell counts between normal and GDM pregnancy. The normal pregnancy group (n = 4 biological replicates) was compared to the GDM pregnancy group (n = 3 biological replicates). Each biological replicate represents an independent human donor. Data are presented as mean ± SEM. Normally distributed data were analysed using a two-sided unpaired Student's t-test; non-parametric data were analysed using a two-sided Mann–Whitney test. Exact p-values for each comparison are shown in the figure. Statistical significance was defined as $P < 0.05$.

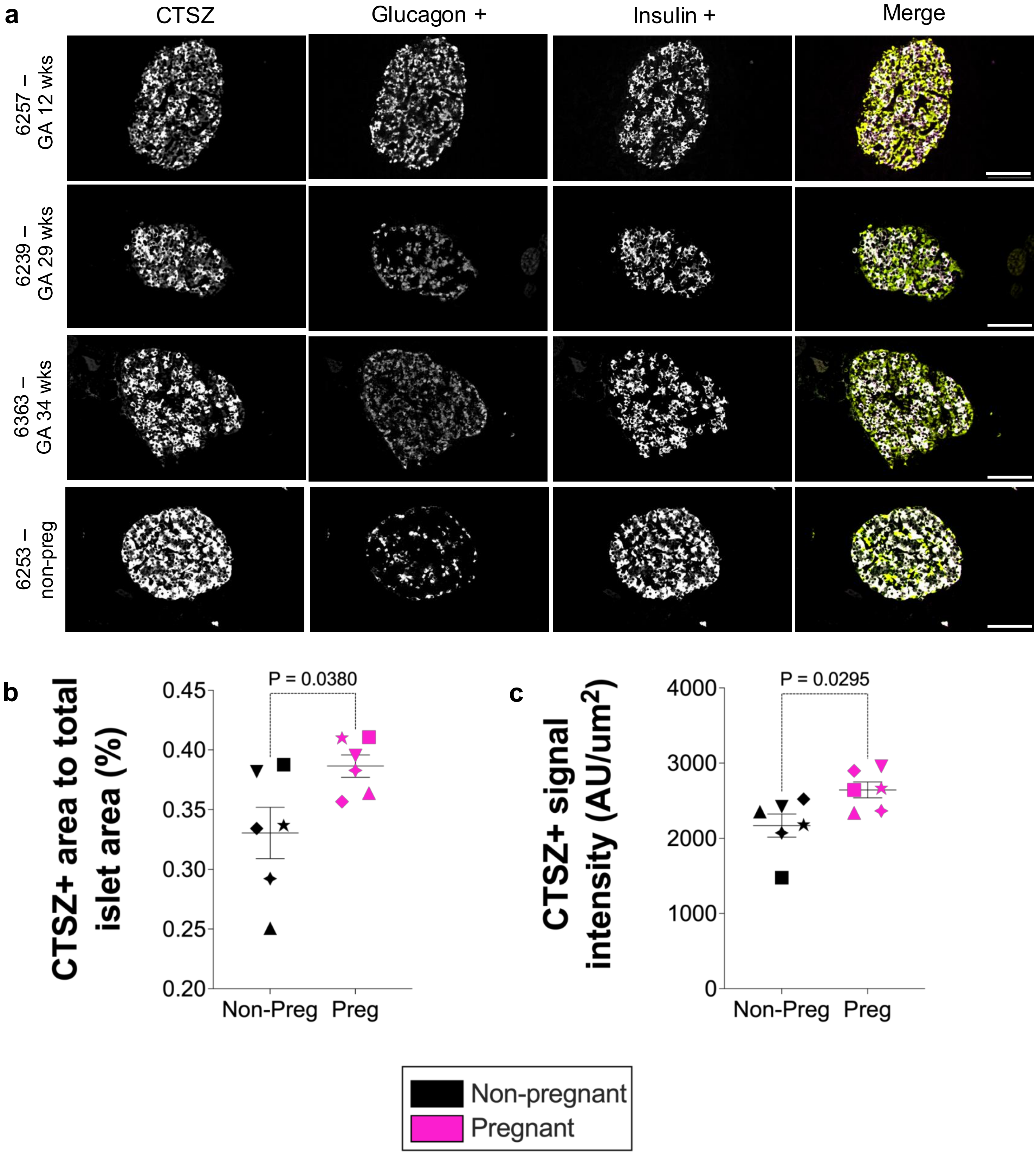
a
CTSZ
Glucagon +
Insulin +
Merge
6257 – GA 12 wks
6239 – GA 29 wks
6363 – GA 34 wks
6253 – non-preg
b
CTSZ+ area to total islet area (%)
P = 0.0380
0.45
0.40
0.35
0.30
0.25
0.20
Non-Preg
Preg
c
CTSZ+ signal intensity (AU/um^2)
P = 0.0295
4000
3000
2000
1000
0
Non-Preg
Preg
Non-pregnant
Pregnant

Fig. S6: Upregulation of the Cathepsin Z (CTSZ) receptor in pancreatic islets during pregnancy. **a** Immunofluorescence of human pancreatic sections (IHC-IF) from pregnant women at different gestational ages. CTSZ signal is observed in islets, overlapping with insulin. The grayscale images represent the individual channels for the CTSZ and insulin. The composite merged image displays CTSZ (green) and glucagon (yellow), and insulin (magenta). Scale bar = 200 μm. Representative images from four donors are shown; similar results were observed across the 12 biological replicates, each representing an independent human donor. Quantitative comparisons of CTSZ between pregnant and non-pregnant women. The data includes comparisons for the **b** proportions of CTSZ positive area relative to whole islets (measured area as a percentage of total whole islet area) and **c** signal intensity of CTSZ detected in whole islets of pregnant women compared to non-pregnant controls. Symbols in each figure correspond to individual donors as indicated in Table S1. The pregnant group (n = 6 biological replicates) was compared to the non-pregnant control group (n = 6 biological replicates). Each biological replicate represents an independent human donor. Data are presented as mean ± SEM. Normally distributed data were analysed using a two-sided unpaired Student's t-test; non-parametric data were analysed using a two-sided Mann–Whitney test. Exact p-values for each comparison are shown in the figure. Statistical significance was defined as $P < 0.05$. * GA – gestational age, wks – weeks.

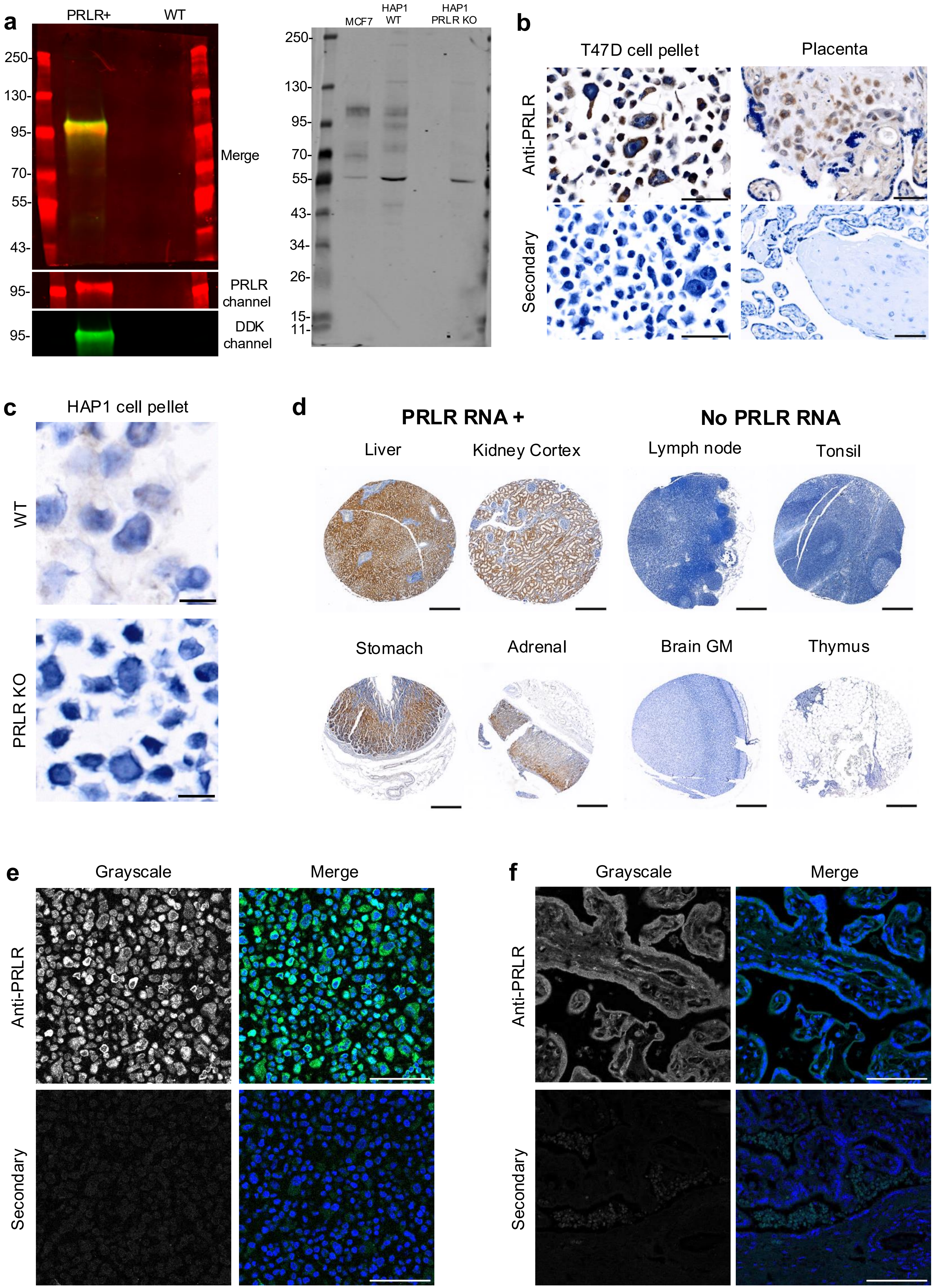
a
PRLR+
WT
250-
130-
95-
70-
55-
43-
Merge
95-
PRLR channel
95-
DDK channel
MCF7
HAP1 WT
HAP1 PRLR KO
250-
130-
95-
70-
55-
43-
34-
26-
15-
11-
b
T47D cell pellet
Placenta
Anti-PRLR
Secondary
c
HAP1 cell pellet
WT
PRLR KO
d
PRLR RNA +
No PRLR RNA
Liver
Kidney Cortex
Lymph node
Tonsil
Stomach
Adrenal
Brain GM
Thymus
e
Grayscale
Merge
Anti-PRLR
Secondary
f
Grayscale
Merge
Anti-PRLR
Secondary

Supp. Fig. S7: Validation of anti-prolactin receptor (PRLR) antibody.
a Immunoblot using the anti-PRLR antibody on PRLR overexpression in HEK293T cell lysate. Bands are observed in the PRLR overexpression cell lysate at the predicted molecular weight (PRLR with DDK-tag) but not in the wild-type (WT) HEK293T cell lysate. A second immunoblot using the anti-PRLR antibody on MCF7 cells, HAP1 WT, and HAP1 PRLR knockout (KO) cell lysate. Bands are observed at the predicted molecular weight for PRLR in MCF7 and HAP1 WT lysates but are absent in the HAP1 PRLR KO lysate. **b** FFPE T47D cell pellets and placental tissue labelled with the anti-PRLR antibody show a positive signal. No signal is observed in the secondary-only control. Scale bar = 50 µm. **c** IHC of HAP1 WT and PRLR KO Cells. Formalin-fixed paraffin-embedded (FFPE) HAP1 WT and PRLR KO cell pellets labelled with the anti-PRLR antibody show a positive signal in WT cells and no signal in PRLR KO cells. Scale bar = 10 µm. **d** IHC analysis using the anti-PRLR antibody on human tissues known to express PRLR RNA and tissues where PRLR RNA is absent. Tissues with PRLR expression exhibit a positive signal, while those without PRLR RNA show no signal. Scale bar = 500 µm. **e** IHC-IF of FFPE T47D cell pellets and **f** placental tissue labelled with the anti-PRLR antibody showing a positive signal. No signal is observed in the secondary-only control. Scale bars: T47D cell pellets and placental tissue = 100 µm.

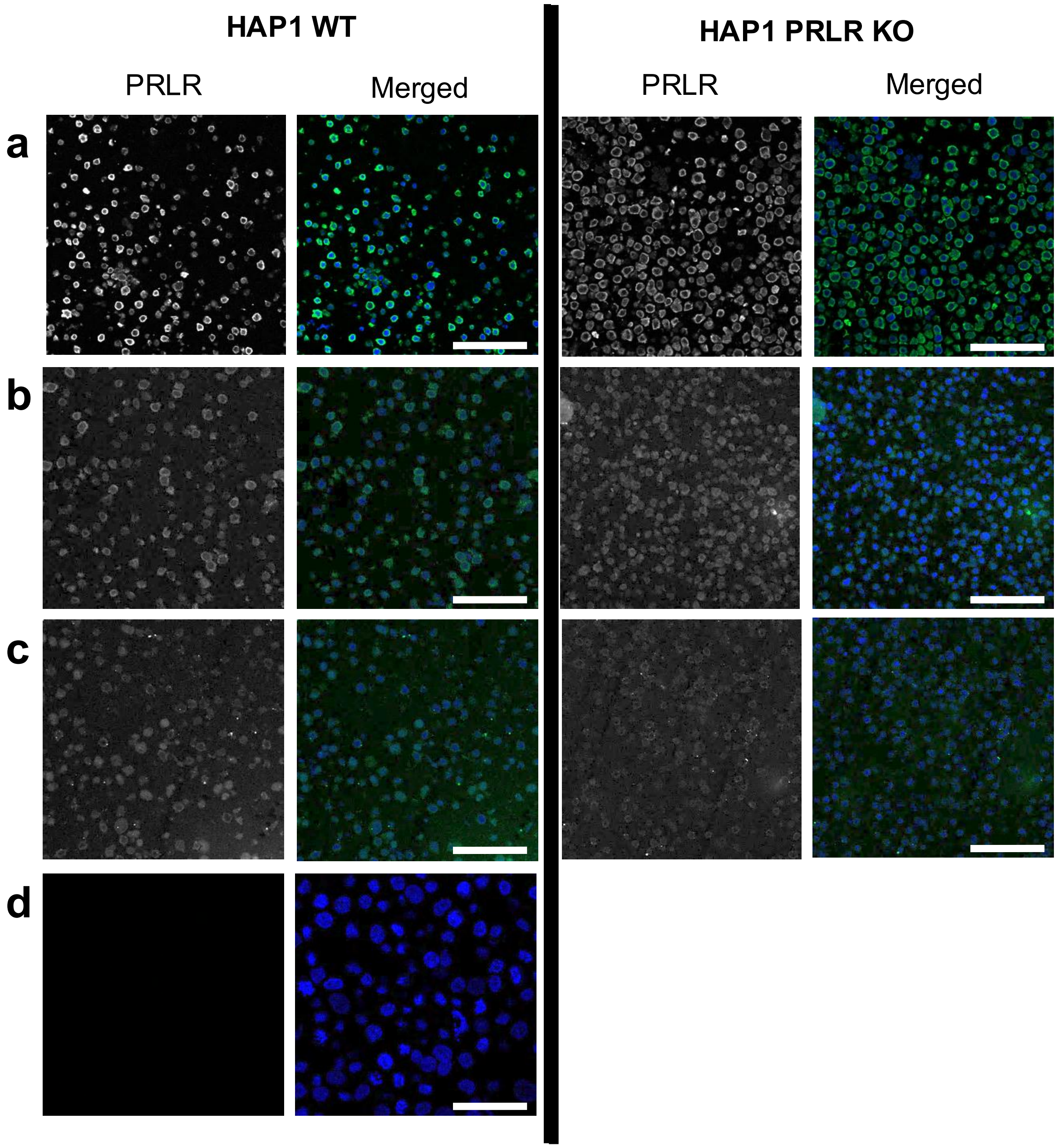

Supp. Fig. S8: Assessment of specificity of anti-prolactin receptor (PRLR) antibodies by IHC-IF of FFPE HAP 1 wild-type (WT) and *PRLR* knockout (KO) cell pellets.

Antibodies that were noted to label both HAP1 WT and *PRLR* KO cells are shown. These include **a** PRLR monoclonal antibody (Proteintech, cat. no. 67292), **b** Prolactin R antibody B6.2 + PRLR742 (Novus biologicals, cat. no. NBP2-34286), **c** Human Prolactin R Antibody (R&D systems, cat. no. MAB1167), **d** Secondary only control. Grayscale images of the PRLR channel is shown and the merged image shows the nuclei (DAPI, blue) and PRLR (green). Scale bar = 100 µm.

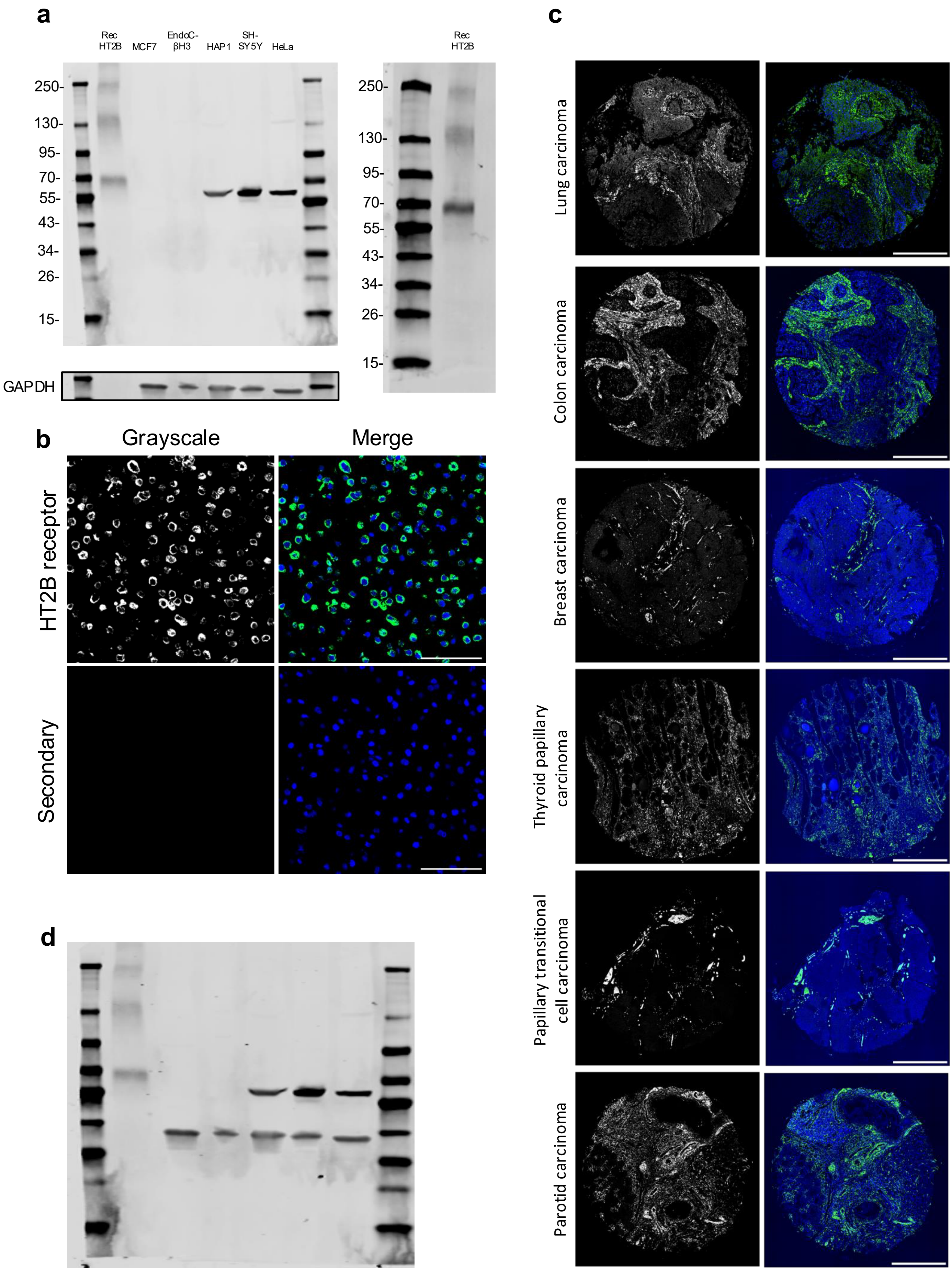
a
Rec HT2B
MCF7
EndoC-βH3
HAP1
SH-SY5Y
HeLa
250-
130-
95-
70-
55-
43-
34-
26-
15-
GAPDH
Rec HT2B
b
Grayscale
Merge
HT2B receptor
Secondary
c
Lung carcinoma
Colon carcinoma
Breast carcinoma
Thyroid papillary carcinoma
Papillary transitional cell carcinoma
Parotid carcinoma
d

Supp. Fig. S9: Anti-serotonin 2B (5-HT2B) receptor antibody validation.

a Immunoblot using the anti-5-HT2B receptor antibody of full-length human 5-HT2B recombinant protein and whole cell lysates. Bands are observed in lanes containing full-length recombinant protein and whole cell lysates from cells known to express the HTR2B gene (HAP1, SH-SY5Y, and HeLa). No bands are seen in lysates where HTR2B receptor is absent (MCF7 and EndoC-βH3). GAPDH is used as a loading control. A second immunoblot of full-length human 5-HT2B recombinant protein using the anti-5-HT2B antibody. **b** Formalin-fixed paraffin-embedded (FFPE) HAP1 wild-type cell pellets labelled with the anti-5-HT2B receptor antibody show positive staining. No staining is observed in the secondary-only control. Images include grayscale of the 5-HT2B receptor channel and the merged image (5-HT2B and DAPI). Scale bar = 100 µm. **c** IHC-IF analysis of human cancer tissues shows labelling by the anti-5-HT2B receptor antibody in malignancies known to express the 5-HT2B receptor. Grayscale images of the 5-HT2B receptor channel and merged images (DAPI and 5-HT2B) are shown. Scale bar = 500 µm. **d** The uncropped immunoblot shown in panel **a** following reprobing with GAPDH as a loading control.

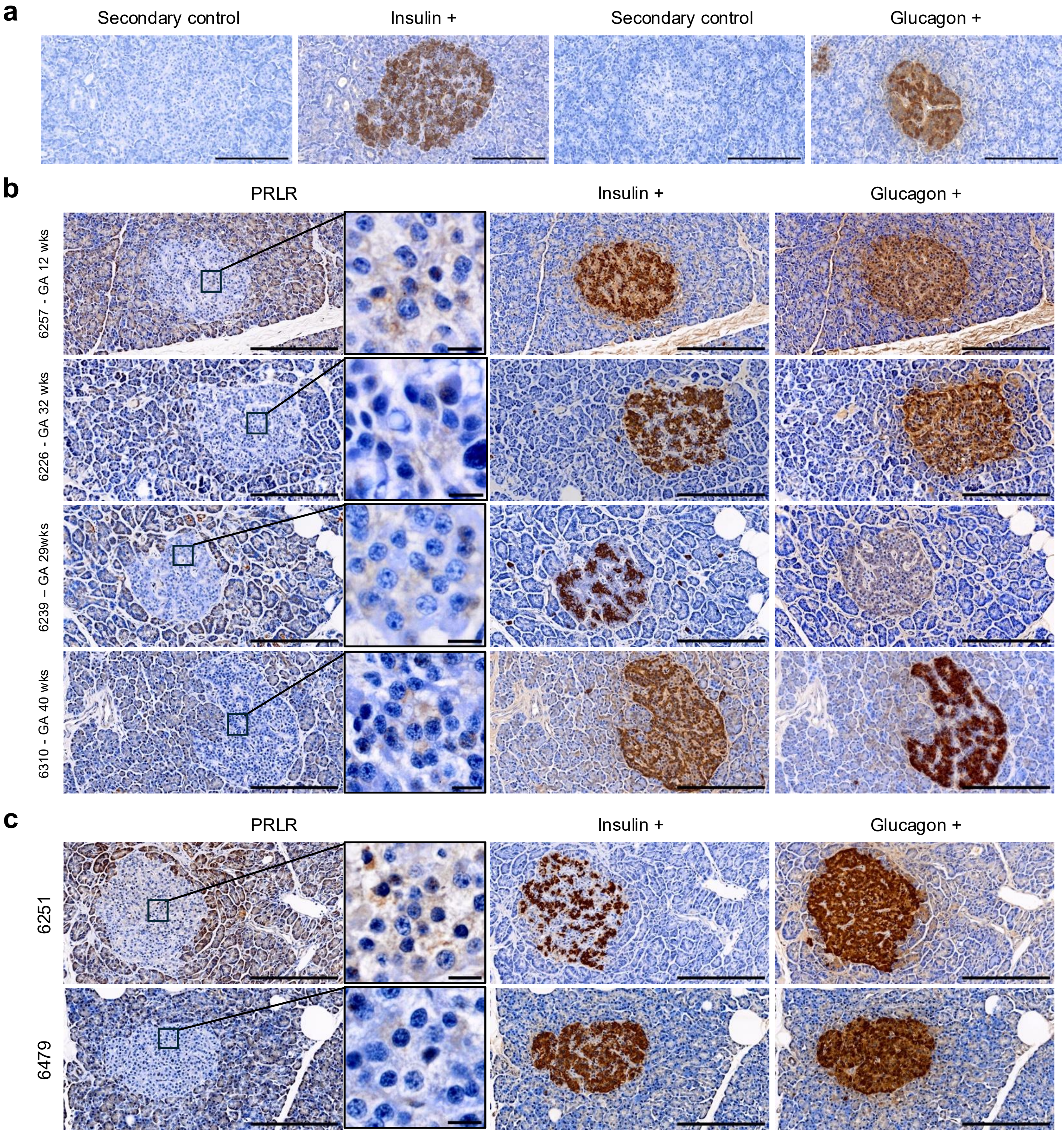

Supp. Fig. S10: Immunohistochemistry (IHC) labelling of prolactin receptor (PRLR) in pancreatic islets in pregnancy and non-pregnant controls.
a Secondary only controls show no detectable signal in islets. Serial sections labelled with the secondary control followed by either insulin or glucagon are shown. **b** PRLR signal is detected in islets from pregnant women. **c** PRLR signal is detected in islets from non-pregnant controls.
For **b** and **c** serial sections are shown labelled for PRLR, insulin and glucagon. Scale bar = 1000µm and 5µm for inset image. Representative images from six donors are shown; similar results were observed across the 14 biological replicates, each representing an independent human donor. *GA – gestational age, wks – weeks.

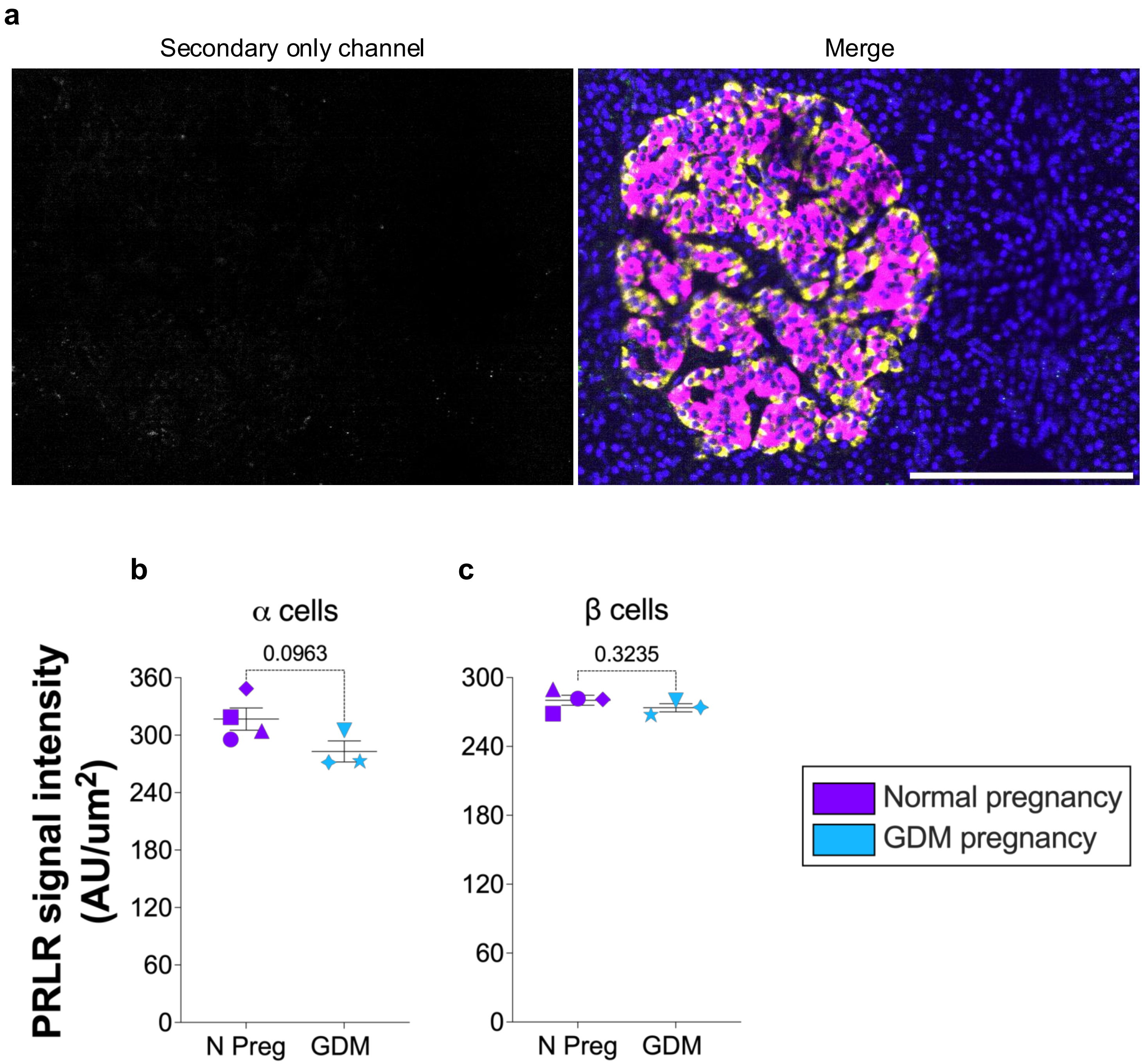

Supp. Fig. S11: Secondary only controls of pancreatic tissue labelled by immunofluorescence (IHC-IF) and prolactin receptor (PRLR) expression in gestational diabetes (GDM) compared to normal pregnancy.

a IHC-IF of a human pancreatic section. The grayscale image represents the secondary control where only the VectaFluor™ Excel amplified anti-rabbit secondary antibody was applied to the tissue section, without the primary anti-PRLR antibody. The composite merged mage is shown in colour (secondary only in green (not seen), nuclei (DAPI) in blue, α cells in yellow, and β cells in magenta). Scale bar = 200 µm. Signal intensity of PRLR detected in **b** α and **c** β cells of normal compared to GDM pregnancy. The normal pregnancy group (n = 4 biological replicates) was compared to the GDM pregnancy group (n = 3 biological replicates). Each biological replicate represents an independent human donor. Data are presented as mean ± SEM. Normally distributed data were analysed using a two-sided unpaired Student's t-test; non-parametric data were analysed using a two-sided Mann–Whitney test. Exact p-values for each comparison are shown in the figure. Statistical significance was defined as $P < 0.05$.

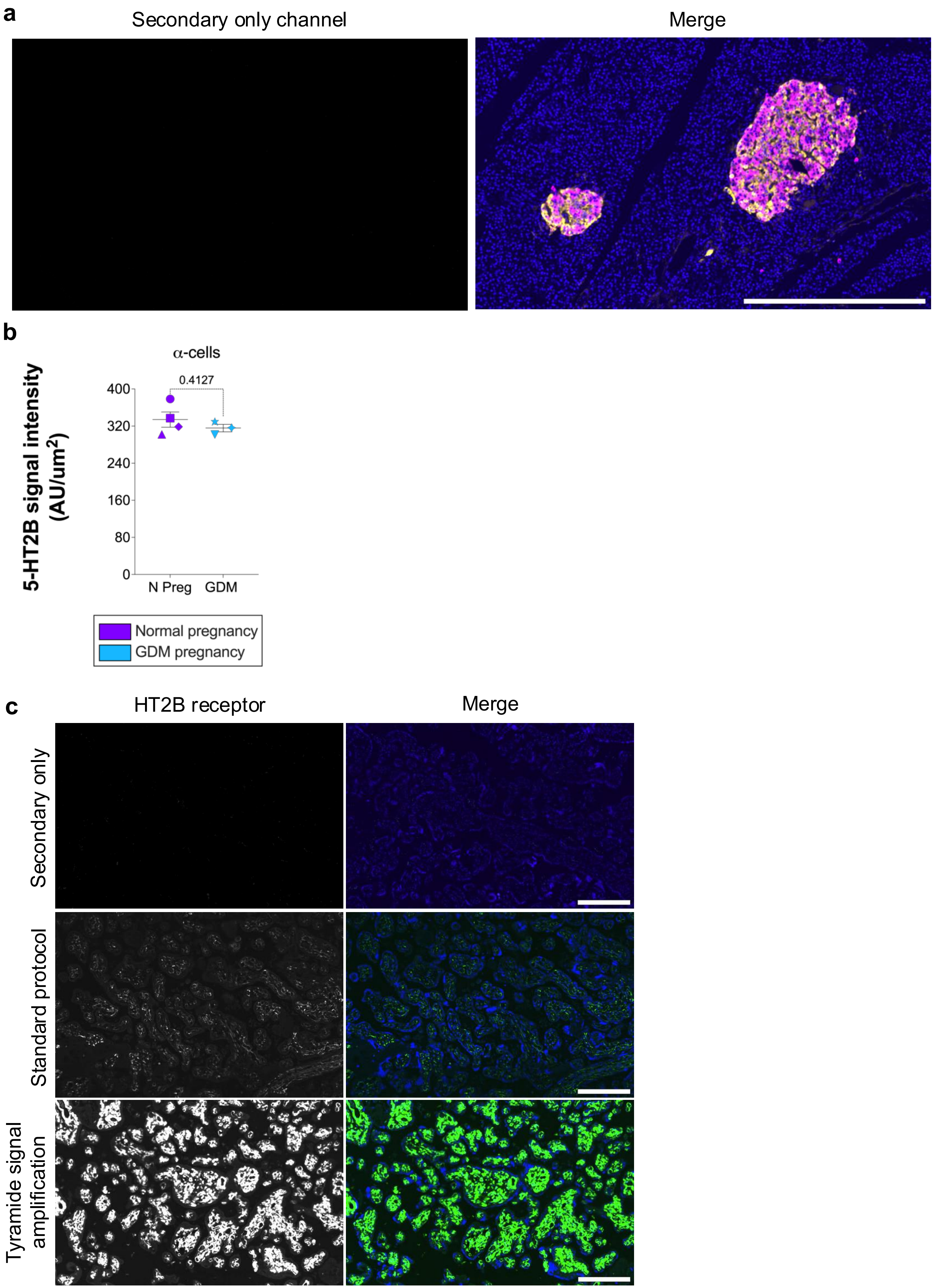
a
Secondary only channel
Merge
b
α-cells
5-HT2B signal intensity (AU/um2)
0.4127
400
320
240
160
80
0
N Preg
GDM
Normal pregnancy
GDM pregnancy
c
HT2B receptor
Merge
Secondary only
Standard protocol
Tyramide signal amplification

Supp. Fig. S12: Secondary only controls, serotonin 2B (5-HT2B) receptor expression in gestational diabetes (GDM) compared to normal pregnancy, and validation of the efficacy of tyramide signal amplification.

a Immunofluorescence (IHC-IF) with tyramide signal amplification of a human pancreatic section. The grayscale image represents the secondary control where only the Alexa Fluor™ 488 Tyramide SuperBoost™ Kit, goat anti-mouse IgG, was applied to the tissue section in the absence of the primary anti-HT2B receptor antibody. The composite merged mage is shown in colour (secondary only in green (not seen), nuclei (DAPI) in blue, α cells in yellow, and β cells in magenta). Scale bar = 200 μm.

b Signal intensity of the 5-HT2B receptor detected in α-cells of women with normal pregnancy compared to GDM pregnancy. The normal pregnancy group (n = 4 biological replicates) was compared to the GDM pregnancy group (n = 3 biological replicates). Each biological replicate represents an independent human donor. Data are presented as mean ± SEM. Normally distributed data were analysed using a two-sided unpaired Student’s t-test; non-parametric data were analysed using a two-sided Mann–Whitney test. Exact p-values for each comparison are shown in the figure. Statistical significance was defined as $P < 0.05$. **c** Immunofluorescence (IHC-IF) of human placental sections. Human placental sections were labelled using a standard IHC-IF protocol and tyramide signal amplification. A secondary-only control is also shown. Tyramide signal amplification provides enhanced detection of the 5-HT2B receptor compared to the standard protocol. Consistent antibody concentrations and identical image acquisition settings—including magnification, resolution, and light intensity—were maintained across all conditions. Grayscale images represent the 5-HT2B receptor channel, while the composite merged images show the 5-HT2B receptor in green and nuclei (DAPI) in blue. Scale bar = 100 μm.

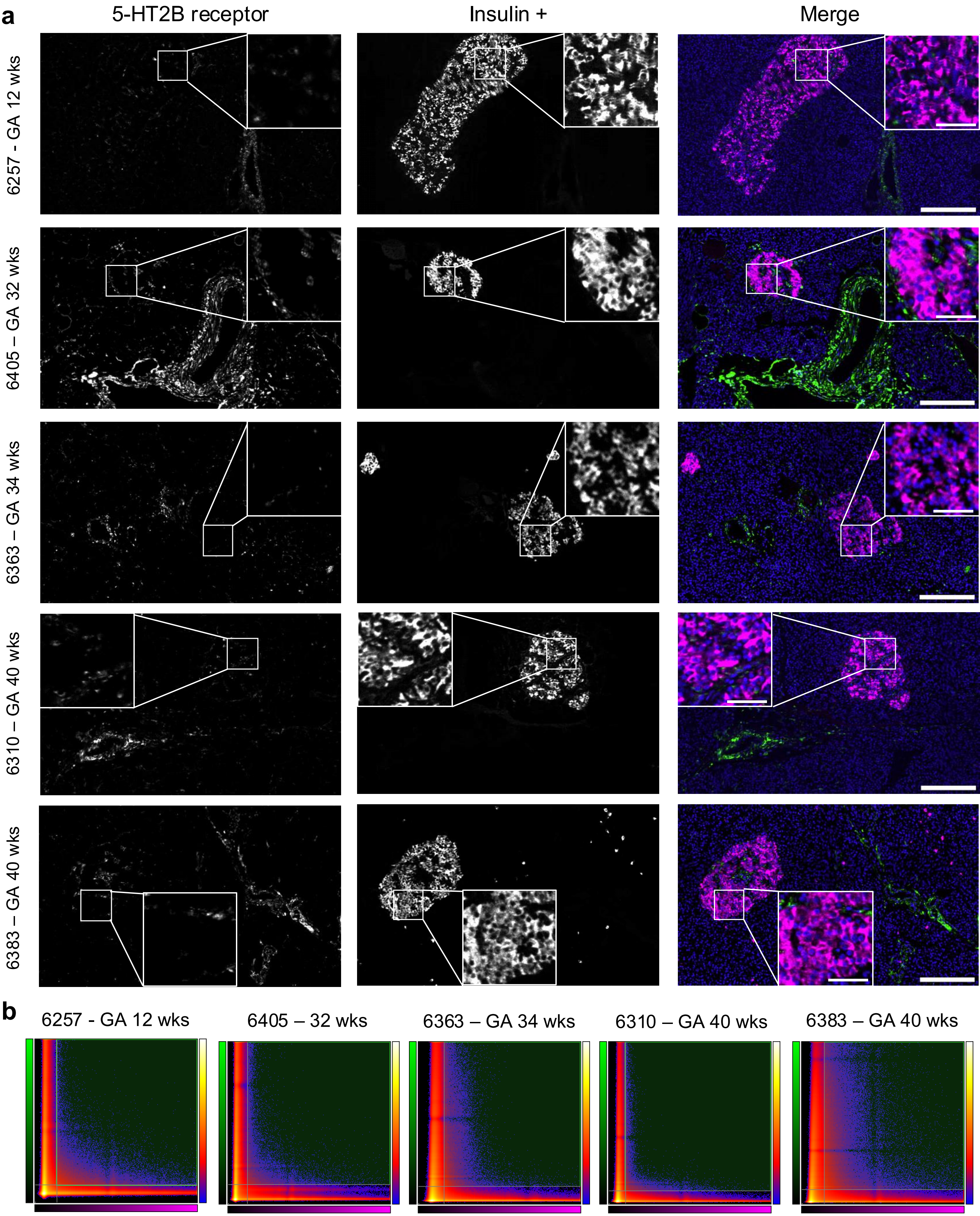

c

	6257–GA 12wks	6405–GA 32wks	6363–GA 34wks	6310–GA 40wks	6383–GA 40wks
M1	0.0137	0.0045	0.006	0.0017	0.0256
M2	0.0023	0.0017	0.0193	0.0052	0.0489

Supp. Fig. S13: Absence of serotonin 2 (5-HT2B) receptor in insulin-producing pancreatic β cells during pregnancy.

a Immunofluorescence with tyramide signal amplification of human pancreatic sections (IHC-IF) from pregnant women at different gestational ages. No staining of the 5-HT2B receptor is observed within β cells; however, 5-HT2B receptor signal within pancreatic ducts is evident. The grayscale images represent the individual channels for the 5-HT2B receptor and insulin. The composite merged image displays the 5-HT2B receptor (green) and insulin (magenta) channels. Scale bar = 200 µm for the image overview and 50 µm for the magnified area in the inset. Representative images from five donors are shown; similar results were observed across the 14 biological replicates, each representing an independent human donor. **b** Colocalisation analysis demonstrating no colocalisation between the 5-HT2B receptor and insulin signals. Scatter plots showing pixel intensity from the 5-HT2B receptor and insulin channels plotted against each other are presented. Pixel intensity for each channel is plotted on the respective axes: the 5-HT2B receptor channel is represented in green on the y-axis, and the insulin channel is represented in magenta on the x-axis. Whole tissue sections from pregnant women at different gestational ages were analysed. Scatter plots from five donors are shown; similar results were observed across the 14 biological replicates, each representing an independent human donor. **c** Mander's overlap coefficients are shown in the table for each whole tissue section, where M1 represents the overlap of the insulin signal over the 5-HT2B receptor signal, and M2 represents the 5-HT2B receptor signal overlapping the insulin signal. Mander's overlap coefficients from five donors are shown; similar results were observed across the 14 biological replicates, each representing an independent human donor. * GA – gestational age, wks – weeks.

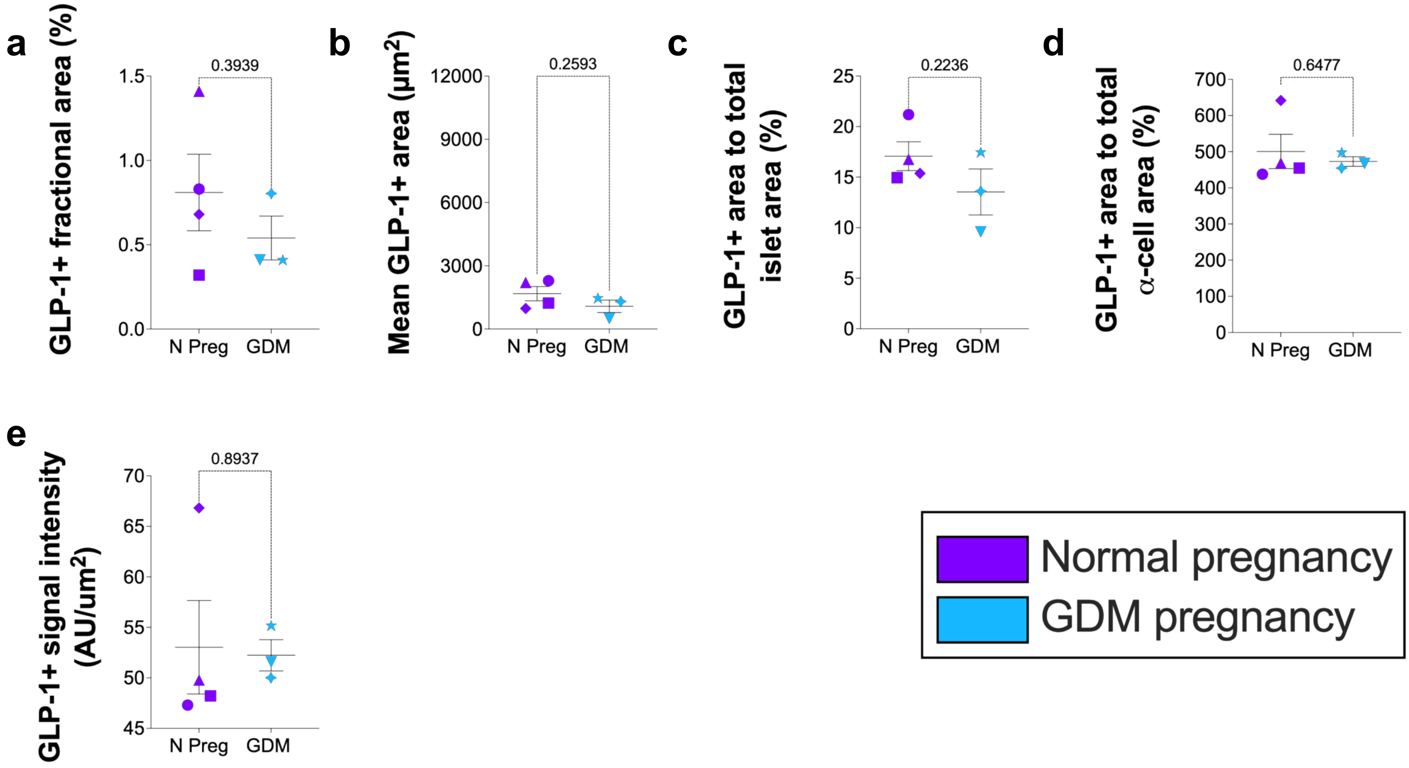

Supp. Fig. S14: Quantitative comparisons of glucagon-like peptide-1 (GLP-1) area and signal intensity between normal and gestational diabetes (GDM) pregnancy.
a-e The data includes comparisons for **a** fractional and **b** mean area of GLP-1 positive area. Additionally, comparisons of the proportions of GLP-1 positive area relative to **c** whole islets (measured area as a percentage of total whole islet area) and **d** relative to α cell area (measured area as a percentage of α cell area) are shown. **e** Signal intensity of GLP-1 detected in α cells of GDM pregnancy compared to normal pregnancy controls. Symbols in each figure correspond to individual donors as indicated in Table 1. The normal pregnancy group (n = 4 biological replicates) was compared to the GDM pregnancy group (n = 3 biological replicates). Each biological replicate represents an independent human donor. Data are presented as mean ± SEM. Normally distributed data were analysed using a two-sided unpaired Student’s t-test; non-parametric data were analysed using a two-sided Mann–Whitney test. Exact p-values for each comparison are shown in the figure. Statistical significance was defined as $P < 0.05$.
