# Supplementary material for: A new paradigm of islet adaptations in human pregnancy: insights from immunohistochemistry and proteomics
Source: Nat Commun. 2025 Jul 21;16:6687. doi: 10.1038/s41467-025-61852-5 (PMC12280027; doi:10.1038/s41467-025-61852-5)
Supplement: Supplementary file 2 — Description of Additional Supplementary Files [file 41467_2025_61852_MOESM2_ESM.pdf]

Description of Additional Supplementary Files

Supplementary Data S1: Descriptive characteristics of women who donated their pancreata.

Supplementary Data S2: List of antibodies

Supplementary Data S3: Raw intensities for proteins detected by liquid chromatography mass spectometry (LC-MS/MS). Data beloging to tissue from each donor for isolated islets and exocrine tissue is shown. *(Exo - exocrine, C - non-pregnant control, P - pregnant)
