# Supplementary material for: A new paradigm of islet adaptations in human pregnancy: insights from immunohistochemistry and proteomics
Source: Nat Commun. 2025 Jul 21;16:6687. doi: 10.1038/s41467-025-61852-5 (PMC12280027; doi:10.1038/s41467-025-61852-5)
Supplement: Supplementary file 3 — Supplementary Data S1 [file 41467_2025_61852_MOESM3_ESM.pptx]

## Slide 1
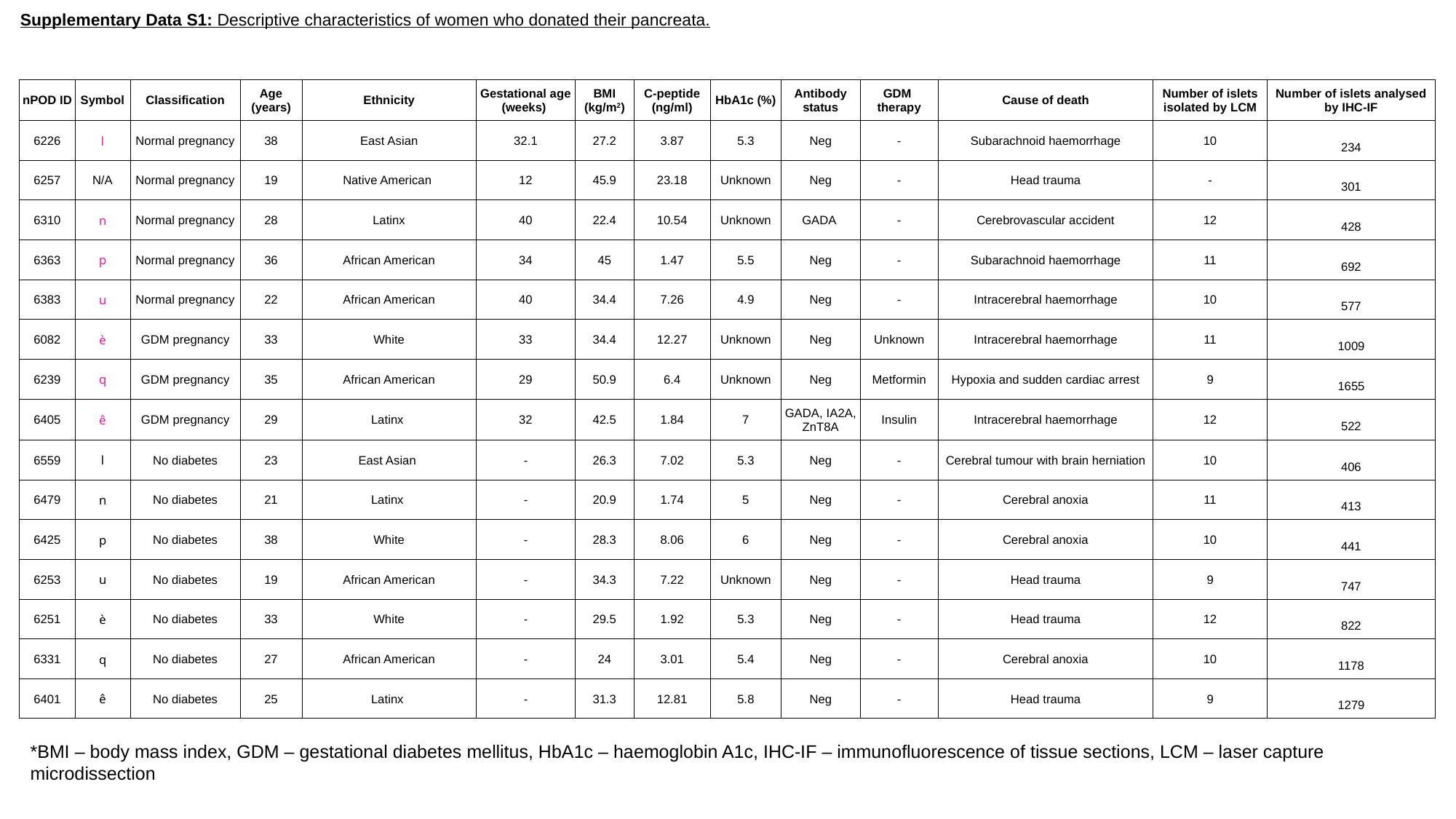

| Supplementary Data S1: Descriptive characteristics of women who donated their pancreata. | | | | | | | | | | | | | |
| --- | --- | --- | --- | --- | --- | --- | --- | --- | --- | --- | --- | --- | --- |
| | | | | | | | | | | | | | |
| nPOD ID | Symbol | Classification | Age (years) | Ethnicity | Gestational age (weeks) | BMI (kg/m2) | C-peptide (ng/ml) | HbA1c (%) | Antibody status | GDM therapy | Cause of death | Number of islets isolated by LCM | Number of islets analysed by IHC-IF |
| 6226 | l | Normal pregnancy | 38 | East Asian | 32.1 | 27.2 | 3.87 | 5.3 | Neg | - | Subarachnoid haemorrhage | 10 | 234 |
| 6257 | N/A | Normal pregnancy | 19 | Native American | 12 | 45.9 | 23.18 | Unknown | Neg | - | Head trauma | - | 301 |
| 6310 | n | Normal pregnancy | 28 | Latinx | 40 | 22.4 | 10.54 | Unknown | GADA | - | Cerebrovascular accident | 12 | 428 |
| 6363 | p | Normal pregnancy | 36 | African American | 34 | 45 | 1.47 | 5.5 | Neg | - | Subarachnoid haemorrhage | 11 | 692 |
| 6383 | u | Normal pregnancy | 22 | African American | 40 | 34.4 | 7.26 | 4.9 | Neg | - | Intracerebral haemorrhage | 10 | 577 |
| 6082 | è | GDM pregnancy | 33 | White | 33 | 34.4 | 12.27 | Unknown | Neg | Unknown | Intracerebral haemorrhage | 11 | 1009 |
| 6239 | q | GDM pregnancy | 35 | African American | 29 | 50.9 | 6.4 | Unknown | Neg | Metformin | Hypoxia and sudden cardiac arrest | 9 | 1655 |
| 6405 | ê | GDM pregnancy | 29 | Latinx | 32 | 42.5 | 1.84 | 7 | GADA, IA2A, ZnT8A | Insulin | Intracerebral haemorrhage | 12 | 522 |
| 6559 | l | No diabetes | 23 | East Asian | - | 26.3 | 7.02 | 5.3 | Neg | - | Cerebral tumour with brain herniation | 10 | 406 |
| 6479 | n | No diabetes | 21 | Latinx | - | 20.9 | 1.74 | 5 | Neg | - | Cerebral anoxia | 11 | 413 |
| 6425 | p | No diabetes | 38 | White | - | 28.3 | 8.06 | 6 | Neg | - | Cerebral anoxia | 10 | 441 |
| 6253 | u | No diabetes | 19 | African American | - | 34.3 | 7.22 | Unknown | Neg | - | Head trauma | 9 | 747 |
| 6251 | è | No diabetes | 33 | White | - | 29.5 | 1.92 | 5.3 | Neg | - | Head trauma | 12 | 822 |
| 6331 | q | No diabetes | 27 | African American | - | 24 | 3.01 | 5.4 | Neg | - | Cerebral anoxia | 10 | 1178 |
| 6401 | ê | No diabetes | 25 | Latinx | - | 31.3 | 12.81 | 5.8 | Neg | - | Head trauma | 9 | 1279 |
*BMI – body mass index, GDM – gestational diabetes mellitus, HbA1c – haemoglobin A1c, IHC-IF – immunofluorescence of tissue sections, LCM – laser capture microdissection
