# Supplementary material for: A new paradigm of islet adaptations in human pregnancy: insights from immunohistochemistry and proteomics
Source: Nat Commun. 2025 Jul 21;16:6687. doi: 10.1038/s41467-025-61852-5 (PMC12280027; doi:10.1038/s41467-025-61852-5)
Supplement: Supplementary file 4 — Supplementary Data S2 [file 41467_2025_61852_MOESM4_ESM.pptx]

## Slide 1
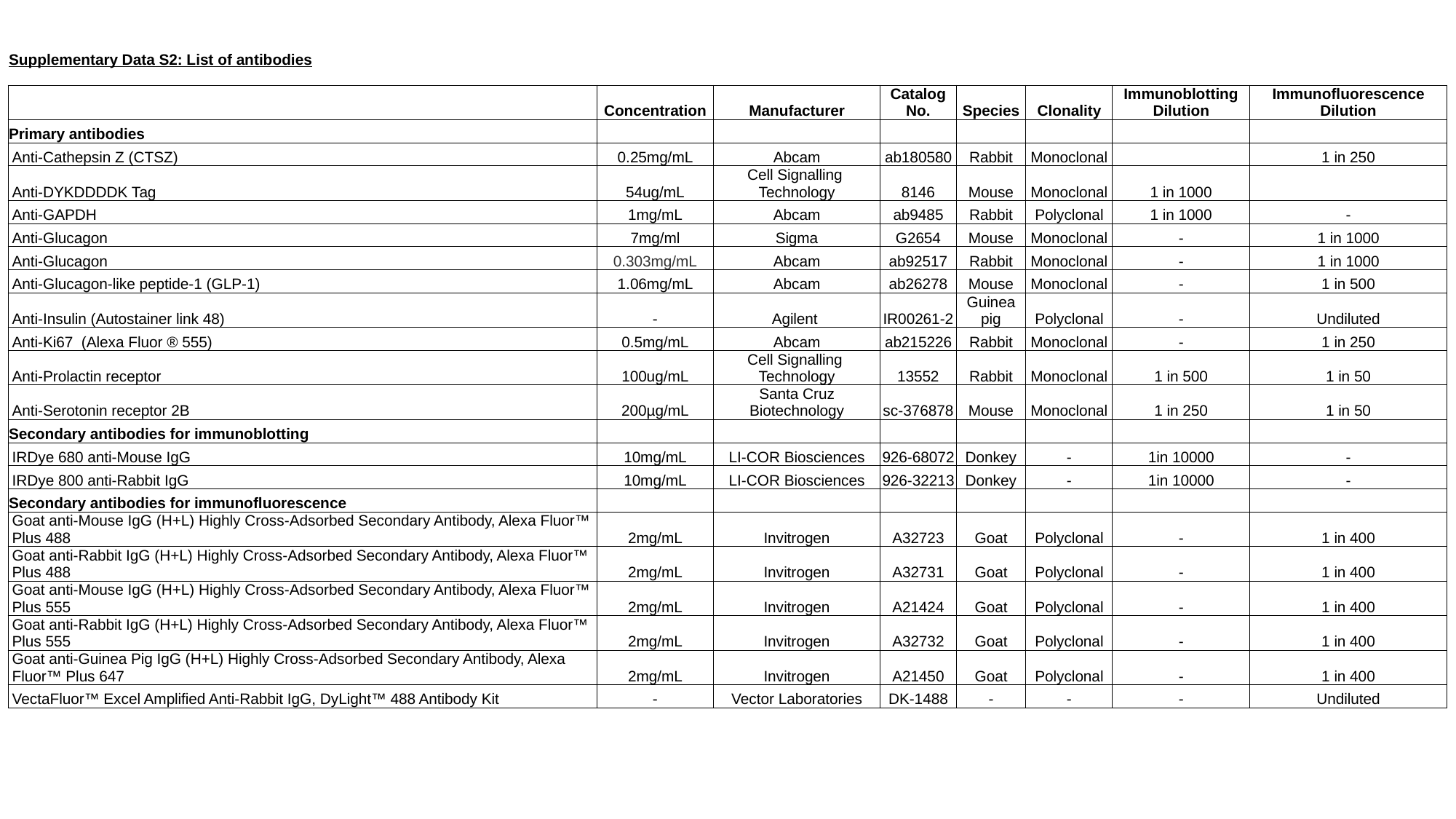

| Supplementary Data S2: List of antibodies | | | | | | | |
| --- | --- | --- | --- | --- | --- | --- | --- |
| | Concentration | Manufacturer | Catalog No. | Species | Clonality | Immunoblotting Dilution | Immunofluorescence Dilution |
| Primary antibodies | | | | | | | |
| Anti-Cathepsin Z (CTSZ) | 0.25mg/mL | Abcam | ab180580 | Rabbit | Monoclonal | | 1 in 250 |
| Anti-DYKDDDDK Tag | 54ug/mL | Cell Signalling Technology | 8146 | Mouse | Monoclonal | 1 in 1000 | |
| Anti-GAPDH | 1mg/mL | Abcam | ab9485 | Rabbit | Polyclonal | 1 in 1000 | - |
| Anti-Glucagon | 7mg/ml | Sigma | G2654 | Mouse | Monoclonal | - | 1 in 1000 |
| Anti-Glucagon | 0.303mg/mL | Abcam | ab92517 | Rabbit | Monoclonal | - | 1 in 1000 |
| Anti-Glucagon-like peptide-1 (GLP-1) | 1.06mg/mL | Abcam | ab26278 | Mouse | Monoclonal | - | 1 in 500 |
| Anti-Insulin (Autostainer link 48) | - | Agilent | IR00261-2 | Guinea pig | Polyclonal | - | Undiluted |
| Anti-Ki67 (Alexa Fluor ® 555) | 0.5mg/mL | Abcam | ab215226 | Rabbit | Monoclonal | - | 1 in 250 |
| Anti-Prolactin receptor | 100ug/mL | Cell Signalling Technology | 13552 | Rabbit | Monoclonal | 1 in 500 | 1 in 50 |
| Anti-Serotonin receptor 2B | 200µg/mL | Santa Cruz Biotechnology | sc-376878 | Mouse | Monoclonal | 1 in 250 | 1 in 50 |
| Secondary antibodies for immunoblotting | | | | | | | |
| IRDye 680 anti-Mouse IgG | 10mg/mL | LI-COR Biosciences | 926-68072 | Donkey | - | 1in 10000 | - |
| IRDye 800 anti-Rabbit IgG | 10mg/mL | LI-COR Biosciences | 926-32213 | Donkey | - | 1in 10000 | - |
| Secondary antibodies for immunofluorescence | | | | | | | |
| Goat anti-Mouse IgG (H+L) Highly Cross-Adsorbed Secondary Antibody, Alexa Fluor™ Plus 488 | 2mg/mL | Invitrogen | A32723 | Goat | Polyclonal | - | 1 in 400 |
| Goat anti-Rabbit IgG (H+L) Highly Cross-Adsorbed Secondary Antibody, Alexa Fluor™ Plus 488 | 2mg/mL | Invitrogen | A32731 | Goat | Polyclonal | - | 1 in 400 |
| Goat anti-Mouse IgG (H+L) Highly Cross-Adsorbed Secondary Antibody, Alexa Fluor™ Plus 555 | 2mg/mL | Invitrogen | A21424 | Goat | Polyclonal | - | 1 in 400 |
| Goat anti-Rabbit IgG (H+L) Highly Cross-Adsorbed Secondary Antibody, Alexa Fluor™ Plus 555 | 2mg/mL | Invitrogen | A32732 | Goat | Polyclonal | - | 1 in 400 |
| Goat anti-Guinea Pig IgG (H+L) Highly Cross-Adsorbed Secondary Antibody, Alexa Fluor™ Plus 647 | 2mg/mL | Invitrogen | A21450 | Goat | Polyclonal | - | 1 in 400 |
| VectaFluor™ Excel Amplified Anti-Rabbit IgG, DyLight™ 488 Antibody Kit | - | Vector Laboratories | DK-1488 | - | - | - | Undiluted |
