# Supplementary material for: A new paradigm of islet adaptations in human pregnancy: insights from immunohistochemistry and proteomics
Source: Nat Commun. 2025 Jul 21;16:6687. doi: 10.1038/s41467-025-61852-5 (PMC12280027; doi:10.1038/s41467-025-61852-5)
Supplement: Supplementary file 6 — Reporting Summary [file 41467_2025_61852_MOESM6_ESM.pdf]

nature portfolio

Corresponding author(s): Faheem Seedat, M Irina Stefana

Last updated by author(s): 23/5/2025

Reporting Summary

Nature Portfolio wishes to improve the reproducibility of the work that we publish. This form provides structure for consistency and transparency in reporting. For further information on Nature Portfolio policies, see our Editorial Policies and the Editorial Policy Checklist.

Statistics

For all statistical analyses, confirm that the following items are present in the figure legend, table legend, main text, or Methods section.

n/a	Confirmed	
[ ]	[x]	The exact sample size (*n*) for each experimental group/condition, given as a discrete number and unit of measurement
[ ]	[x]	A statement on whether measurements were taken from distinct samples or whether the same sample was measured repeatedly
[ ]	[x]	The statistical test(s) used AND whether they are one- or two-sided<br>*Only common tests should be described solely by name; describe more complex techniques in the Methods section.*
[ ]	[x]	A description of all covariates tested
[ ]	[x]	A description of any assumptions or corrections, such as tests of normality and adjustment for multiple comparisons
[ ]	[x]	A full description of the statistical parameters including central tendency (e.g. means) or other basic estimates (e.g. regression coefficient) AND variation (e.g. standard deviation) or associated estimates of uncertainty (e.g. confidence intervals)
[ ]	[x]	For null hypothesis testing, the test statistic (e.g. F, t, r) with confidence intervals, effect sizes, degrees of freedom and P value noted<br>*Give P values as exact values whenever suitable.*
[x]	[ ]	For Bayesian analysis, information on the choice of priors and Markov chain Monte Carlo settings
[x]	[ ]	For hierarchical and complex designs, identification of the appropriate level for tests and full reporting of outcomes
[x]	[ ]	Estimates of effect sizes (e.g. Cohen's *d*, Pearson's *r*), indicating how they were calculated

Our web collection on statistics for biologists contains articles on many of the points above.

Software and code

Policy information about availability of computer code

Data collection	No commercial, open source or custom code was used to collect data for this study.
Data analysis	The following software were used for data analysis: GraphPad Prism (v 10.4.1), Cytoscape (v 3.10.2), String DB (v 12.0), Perseus (v 2.0.11), ImageStudio (v 5.5.4), Arivis Vision4D (v4.1.2) software.

For manuscripts utilizing custom algorithms or software that are central to the research but not yet described in published literature, software must be made available to editors and reviewers. We strongly encourage code deposition in a community repository (e.g. GitHub). See the Nature Portfolio guidelines for submitting code & software for further information.

Data

Policy information about availability of data

All manuscripts must include a data availability statement. This statement should provide the following information, where applicable:

- Accession codes, unique identifiers, or web links for publicly available datasets
- A description of any restrictions on data availability
- For clinical datasets or third party data, please ensure that the statement adheres to our policy

Data will be made available on request.

Research involving human participants, their data, or biological material

Policy information about studies with human participants or human data. See also policy information about sex, gender (identity/presentation), and sexual orientation and race, ethnicity and racism.

Reporting on sex and gender	As pregnant women were studied, findings only apply to female sex. The assessment of female sex was based on biological attributes. Consent was acquired for all patients when sharing their sex. Consent was acquired by the he Network for Pancreatic Organ donors with Diabetes biorepository from which clinical data and samples were acquired. No comparisons with male sex were performed as this did not have relevance to the design of the study due to the focus on islet alterations during human pregnancy.
Reporting on race, ethnicity, or other socially relevant groupings	Race was categorised as African American, Caucasian, Far-East Asian, and Hispanic, based on classifications provided by the Network for Pancreatic Organ Donors with Diabetes (nPOD) biorepository, from which clinical data and samples were obtained. This classification was not used as a proxy for socioeconomic status. Instead, race was considered essential for optimally matching non-pregnant control participants to pregnant cases, thereby ensuring the robustness of statistical analyses.
Population characteristics	This study included pregnant women and matched non-pregnant controls from the Network for Pancreatic Organ Donors with Diabetes (nPOD) biorepository. Covariate-relevant population characteristics considered in the analysis included maternal age, BMI, gestational age at sample collection, and the presence or absence of gestational diabetes mellitus (GDM). Additionally, race/ethnicity was recorded to ensure appropriate matching between cases and controls. Other clinical parameters, such as glucose tolerance status, HbA1c levels, and insulin sensitivity, were also assessed where available.
Recruitment	ticipant samples were obtained from the Network for Pancreatic Organ Donors with Diabetes (nPOD) biorepository. Given the rarity of pancreatic tissue samples from pregnant women, all available samples, along with the best-matched controls, were included in this study.
Ethics oversight	The South Central – Oxford A Research Ethics Committee (REFS 18/SC/0559) approved this project.

Note that full information on the approval of the study protocol must also be provided in the manuscript.

Field-specific reporting

Please select the one below that is the best fit for your research. If you are not sure, read the appropriate sections before making your selection.

☒ Life sciences ☐ Behavioural & social sciences ☐ Ecological, evolutionary & environmental sciences

For a reference copy of the document with all sections, see nature.com/documents/nr-reporting-summary-flat.pdf

Life sciences study design

All studies must disclose on these points even when the disclosure is negative.

Sample size	Due to the global rarity of high-quality pancreatic tissue samples from pregnant women, which can only be obtained post-mortem, a formal sample size calculation was not feasible. Instead, all available samples from the nPOD biorepository were included, creating the largest dataset of islet data from pregnant women to date.
Data exclusions	For proteomic analyses, a single donor and their matched control were excluded due to contamination of islet samples with non-islet tissue. This is detailed in the manuscript.
Replication	Proteomics data was validated using immunohistochemistry. A consistent computational pipeline was applied across all conditions to ensure robust and reproducible data.
Randomization	amples were categorised based on pregnancy status to define each study group. Pregnant and non-pregnant control samples were matched based on key covariates such as age, BMI, and race/ethnicity to minimise confounding
Blinding	Blinding of investigators was not feasible due to the inherent nature of the study, as pregnancy status and associated clinical characteristics were essential for sample selection and analysis. However, to minimise bias, objective and standardised methodologies were applied across all samples, including the use of a consistent computational pipeline for data analysis.

Reporting for specific materials, systems and methods

We require information from authors about some types of materials, experimental systems and methods used in many studies. Here, indicate whether each material, system or method listed is relevant to your study. If you are not sure if a list item applies to your research, read the appropriate section before selecting a response.

Materials & experimental systems

n/a	Involved in the study
	☒ Antibodies
	☒ Eukaryotic cell lines
☒	☐ Palaeontology and archaeology
☒	☐ Animals and other organisms
☒	☐ Clinical data
☒	☐ Dual use research of concern
☒	☐ Plants

Methods

n/a	Involved in the study
☒	☐ ChIP-seq
☒	☐ Flow cytometry
☒	☐ MRI-based neuroimaging

Antibodies

Antibodies used

Primary Antibodies:
Anti-Cathepsin Z (CTSZ): 0.25 mg/mL, Abcam, ab180580, Rabbit, Monoclonal, Immunoblotting 1:250.
Anti-DYKDDDDK Tag: 54 µg/mL, Cell Signalling Technology, 8146, Mouse, Monoclonal, Immunoblotting 1:1000.
Anti-GAPDH: 1 mg/mL, Abcam, ab9485, Rabbit, Polyclonal, Immunoblotting 1:1000.
Anti-Glucagon: 7 mg/mL, Sigma, G2654, Mouse, Monoclonal, Immunofluorescence 1:1000.
Anti-Glucagon: 0.303 mg/mL, Abcam, ab92517, Rabbit, Monoclonal, Immunofluorescence 1:1000.
Anti-Glucagon-like peptide-1 (GLP-1): 1.06 mg/mL, Abcam, ab26278, Mouse, Monoclonal, Immunofluorescence 1:500.
Anti-Insulin (Autostainer link 48): Agilent, IR00261-2, Guinea pig, Polyclonal, Immunofluorescence Undiluted.
Anti-Ki67 (Alexa Fluor® 555): 0.5 mg/mL, Abcam, ab215226, Rabbit, Monoclonal, Immunofluorescence 1:250.
Anti-Prolactin receptor: 100 µg/mL, Cell Signalling Technology, 13552, Rabbit, Monoclonal, Immunoblotting 1:500, Immunofluorescence 1:50.
Anti-Serotonin receptor 2B: 200 µg/mL, Santa Cruz Biotechnology, sc-376878, Mouse, Monoclonal, Immunoblotting 1:250, Immunofluorescence 1:50.

Secondary Antibodies for Immunoblotting:
IRDye 680 anti-Mouse IgG: 10 mg/mL, LI-COR Biosciences, 926-68072, Donkey, Immunoblotting 1:10000.
IRDye 800 anti-Rabbit IgG: 10 mg/mL, LI-COR Biosciences, 926-32213, Donkey, Immunoblotting 1:10000.

Secondary Antibodies for Immunofluorescence:
Goat anti-Mouse IgG (H+L), Alexa Fluor™ Plus 488: 2 mg/mL, Invitrogen, A32723, Goat, Polyclonal, Immunofluorescence 1:400.
Goat anti-Rabbit IgG (H+L), Alexa Fluor™ Plus 488: 2 mg/mL, Invitrogen, A32731, Goat, Polyclonal, Immunofluorescence 1:400.
Goat anti-Mouse IgG (H+L), Alexa Fluor™ Plus 555: 2 mg/mL, Invitrogen, A21424, Goat, Polyclonal, Immunofluorescence 1:400.
Goat anti-Rabbit IgG (H+L), Alexa Fluor™ Plus 555: 2 mg/mL, Invitrogen, A32732, Goat, Polyclonal, Immunofluorescence 1:400.
Goat anti-Guinea Pig IgG (H+L), Alexa Fluor™ Plus 647: 2 mg/mL, Invitrogen, A21450, Goat, Polyclonal, Immunofluorescence 1:400.
VectaFluor™ Excel Amplified Anti-Rabbit IgG, DyLight™ 488 Antibody Kit: Vector Laboratories, DK-1488, Immunofluorescence Undiluted.

Validation

Anti-Cathepsin Z (CTSZ):
Manufacturer: Suitable for WB, IHC-P and reacts with Human samples.
Aiba Y, Harada K, Ito M, et al. Increased expression and altered localization of cathepsin Z are associated with progression to jaundice stage in primary biliary cholangitis. Sci Rep. 2018;8(1):11808. Published 2018 Aug 7. doi:10.1038/s41598-018-30146-w

Anti-DYKDDDDK Tag:.
Manufacturer: Suitable for all applications and reacts with all species.
Brizzard BL, Chubet RG, Vizard DL. Immunoaffinity purification of FLAG epitope-tagged bacterial alkaline phosphatase using a novel monoclonal antibody and peptide elution. Biotechniques. 1994;16(4):730-735.

Anti-GAPDH:
Manufacturer: Suitable western blotting, IHC and immunofluorescence. Suitable for human and mouse samples.
3282 citations in total, the most recent:
Bartosch AMW, Youth EHH, Hansen S, et al. ZCCHC17 Modulates Neuronal RNA Splicing and Supports Cognitive Resilience in Alzheimer's Disease. J Neurosci. 2024;44(3):e2324222023. Published 2024 Jan 17. doi:10.1523/JNEUROSCI.2324-22.2023

Anti-Glucagon, Sigma, G2654:
Manufacturer: immunohistochemistry (formalin-fixed, paraffin-embedded sections): 1:2,000 using human or animal pancreas
320 citations in total, previously used for a paper in Nature Communications:
Katsumoto K, Yennek S, Chen C, et al. Wnt4 is heterogeneously activated in maturing β-cells to control calcium signaling, metabolism and function. Nat Commun. 2022;13(1):6255. Published 2022 Oct 21. doi:10.1038/s41467-022-33841-5

Anti-Glucagon, Abcam, ab92517:
Manufacturer: Validated for western blotting (WB) and immunohistochemistry (IHC) applications. Suitable for human, mouse, and rat samples.
Over 80 citations, most recent:

Jung SR, Lee JH, Ryu H, Gao Y, Lee J. Lithium and exercise ameliorate insulin-deficient hyperglycemia by independently attenuating pancreatic α-cell mass and hepatic gluconeogenesis. Korean J Physiol Pharmacol. 2024;28(1):31-38. doi:10.4196/kjpp.2024.28.1.31

Anti-Glucagon-like peptide-1 (GLP-1):
Manufacturer: Suitable for IHC-P, sELISA and reacts with Mouse, Human, Synthetic peptide samples.
24 citations, most recent:
Odongo K, Hironao KY, Yamashita Y, Ashida H. Development of sandwich ELISAs for detecting glucagon-like peptide-1 secretion from intestinal L-cells and their application in STC-1 cells and mice. J Clin Biochem Nutr. 2023;72(1):28-38. doi:10.3164/jcbn.22-78

Anti-Insulin (Autostainer link 48):
Manufacturer: Immunohisotchemistry reacts with human samples
Previously used for a paper in Nature Communications
Yeh YT, Sona C, Yan X, et al. Restoration of PITPNA in Type 2 diabetic human islets reverses pancreatic beta-cell dysfunction. Nat Commun. 2023;14(1):4250. Published 2023 Jul 17. doi:10.1038/s41467-023-39978-1

Anti-Ki67 (Alexa Fluor® 555):
Comprehensive validation data is provided in the text, including analyses using cancerous tissues showing Ki67 staining of rapidly dividing cancer tissue.
Manufacturer: Suitable for ICC, Flow Cyt (Intra) and reacts with Human samples.
Ermis M, Antmen E, Kuren O, Demirci U, Hasirci V. A Cell Culture Chip with Transparent, Micropillar-Decorated Bottom for Live Cell Imaging and Screening of Breast Cancer Cells. Micromachines (Basel). 2022;13(1):93. Published 2022 Jan 7. doi:10.3390/mi13010093

Anti-Prolactin receptor:
Comprehensive validation data is provided in the text, including analyses using PRLR WT and knockout HAP-1 cell model, human recombinant proteins, FFPE cell pellets derived from lysates of protein-expressing cells and PRLR knockout cell line, as well as normal and tissues known to express the respective proteins.
Manufacturer: Western blotting and immunohistochemistry, reacts with human samples
5 citations, most recent:
Bellavia D, Costa V, De Luca A, et al. The Binomial "Inflammation-Epigenetics" in Breast Cancer Progression and Bone Metastasis: IL-1β Actions Are Influenced by TET Inhibitor in MCF-7 Cell Line. Int J Mol Sci. 2022;23(23):15422. Published 2022 Dec 6. doi:10.3390/ijms232315422

Anti-Serotonin receptor 2B:
Comprehensive validation data is provided in the text, including analyses using cell lysates known to express the target proteins, human recombinant proteins, FFPE cell pellets derived from lysates of protein-expressing cells, as well as cancerous tissues known to express the respective proteins.
Manufacturer: WB, IP, IF, IHC(P) and ELISA, reacts with human, mouse, and rat samples
4 citations, most recent
Gonzalez-Rothi, E.J., et al. 2024. Prolonged intermittent hypoxia differentially regulates phrenic motor neuron serotonin receptor expression in rats following chronic cervical spinal cord injury. Exp. Neurol. 378: 114808.

Eukaryotic cell lines

Policy information about cell lines and Sex and Gender in Research

Cell line source(s)	HAP1 wild-type (WT) parental cells: Horizon Discovery, cat. no. C631, RRID: CVCL_Y019.<br>HAP1 PRLR knockout (KO) cells (25 bp deletion): Horizon Discovery, cat. no. HZGHC003937c0061, RRID: CVCL_TH11.<br>HeLa cells: European Collection of Authenticated Cell Cultures (ECACC), cat. no. 93021013, RRID: CVCL_0030.<br>SH-SY5Y cells: ECACC, cat. no. 94030304, RRID: CVCL_0019.<br>MCF7 cells: ECACC, cat. no. 86012803, RRID: CVCL_0031.<br>U2OS cells: ECACC, cat. no. 92022711, RRID: CVCL_0042.<br>EndoC-βH3 cells: Human Cell Design, France; conditionally immortalised human β-cell line; male origin.
Authentication	Cell lines were not authenticated.
Mycoplasma contamination	Cell lines were not tested for mycoplasma contamination.
Commonly misidentified lines (See ICLAC register)	None were used.

Plants

Seed stocks	N/A
Novel plant genotypes	N/A
Authentication	N/A
